# Physicochemical Properties and Resistant Starch Content of Corn Tortilla Flours Refrigerated at Different Storage Times

**DOI:** 10.3390/foods9040469

**Published:** 2020-04-09

**Authors:** Isela Rojas-Molina, Monsserrat Mendoza-Avila, María de los Ángeles Cornejo-Villegas, Alicia Del Real-López, Eric Rivera-Muñoz, Mario Rodríguez-García, Elsa Gutiérrez-Cortez

**Affiliations:** 1 Laboratorio de Química Medicinal, Facultad de Química Universidad Autónoma de Querétaro, Cerro de las Campanas S/N, Centro Universitario, Querétaro C.P. 76010, Mexico; jirojasmolina@gmail.com (I.R.-M.); monsse_ph@hotmail.com (M.M.-A.); 2 Doctorado en Ciencias Químico-Biológicas, Facultad de Química, Universidad Autónoma de Querétaro, Cerro de las Campanas s/n, Las Campanas, Santiago de Querétaro C.P. 76010, Mexico; 3 Departamento de Ingeniería y Tecnología, FES-Cuautitlán, Laboratorio de Procesos de Transformación y Tecnologías Emergentes de Alimentos, Universidad Nacional Autónoma de México, Cuautitlán Izcalli, Estado de México C.P. 54714, Mexico; angiecornejo@unam.mx; 4 Departamento de Ingeniería Molecular de Materiales y Departamento de Nanotecnología, Centro de Física Aplicada y Tecnología Avanzada, Universidad Nacional Autónoma de México, Blvd. Juriquilla 3001, Juriquilla, Querétaro C.P. 76230, Mexico; adelreal@unam.mx (A.D.R.-L.); erivera@unam.mx (E.R.-M.); mariorodga@gmail.com (M.R.-G.)

**Keywords:** nixtamalization, corn tortilla flours, friability, resistant starch, refrigeration storage, amylose–lipids complexes

## Abstract

The tortilla is a foodstuff that has a short shelf-life, causing great losses to the industry. The objective of this work was to evaluate, for the first time, the physicochemical properties and resistant starch (RS) content of flours. These were obtained from nixtamalized corn tortillas made with traditional and industrial (commercial) methods, stored at 4 °C for 7, 15, and 30 days. The flours were characterized by measuring particle size distribution, color, water absorption index (WAI), water solubility index (WSI), viscosity, calcium, and RS content. Additionally, chemical proximate analysis, scanning electron microscopy (SEM), and thermal analysis were conducted. Storage at 4 °C increased the friability of tortillas and shifted the particle size distribution toward a greater content of coarse particles in corn tortilla flours. The commercial corn tortilla flours showed higher WAI and WSI values than the traditional corn tortilla flours. On the other hand, the traditional corn tortilla flours exhibited higher RS content values than commercial corn tortilla flours as well as peak viscosity. X-ray diffractograms revealed the presence of amylose-lipid complexes (RS5) in experimental samples. The thermograms evidenced three endotherms corresponding to corn starch gelatinization and melting of type I and type II amylose–lipid complexes.

## 1. Introduction

The dietary change as well as a sedentary lifestyle practiced by the population of countries in development implies the intake of hypercaloric diets, rich in calories and low in vitamins and minerals. These have led to an increase in the prevalence of chronic diseases such as diabetes mellitus and cardiovascular diseases. These conditions are important problems for the health sector [1,2,3]. The food industry offers a wide range of products designed for a wide spectrum of consumers that can help to their individual needs, which include health problems [4]. The development of functional foods that, in addition to their basic nutritional functions, provide physiological benefits and reduce the risk of chronic diseases has led to a growth in the use of new technologies and ingredients [5]. Functional foods contain a component with a positive effect on health as well as eliminating a component with a negative effect on it. One of these added components is resistant starch (RS), which is a functional ingredient, especially in foods with a high content of dietary fiber. These foods are used to prevent various pathologies such as obesity, diabetes, and colon cancer, among others [6].

Studies related to the content of RS in products made from nixtamalized corn have been directed to the effect of this component on serum glucose levels in vivo studies [7,8]. In addition, it has been reported that the content of RS can increase in a food in different ways, for example, during storage [9]. Nevertheless, a process for obtaining products or ingredients with high RS content, obtained from nixtamalized byproducts, has not been proposed yet.

The tortilla is a basic foodstuff in the Mexican population diet, so the tortilla industry annually processes millions of tons of corn for its production [10]. On the other hand, 29% of tortilla production is wasted [11], so it is necessary to reduce product losses that involve energy and raw materials expenditure by reusing sub-products with nutraceutical properties for the benefit of consumer health. Therefore, it is possible to propose an alternative for the manufacture of food products and ingredients with high RS content focused on the needs of the Mexican population, either as a preventive to chronic diseases as well as avoiding waste, ecological damage, and promoting the use of by-products from the tortilla industry. Based on the above, the objective of this work was to evaluate the physicochemical properties and resistant starch content of flours obtained from nixtamalized corn tortillas, considered as waste and lost by the tortilla industry, stored in refrigeration at different periods of time.

## 2. Materials and Methods 

### 2.1. Preparation of Experimental Samples

The nixtamal flour obtained by the traditional method (TF) was prepared with corn (variety “Criollo Acatlán”) by using the traditional nixtamalization method described by Gutiérrez-Cortez et al. [12], where 3 kg of corn were cooked in a solution of 6 L of distilled water and 30 g of calcium hydroxide (reagent powder, Fermont, Monterrey, NL, Mexico). The corn kernels were added into a container and cooked at 92 °C for 25 min. After cooking, the corn was steeped for 9 h. Subsequently, the cooking liquid (nejayote) was drained off and the nixtamal sample (cooked corn kernels) were washed twice in distilled water using 2:1 (*v/w*) ratio by stirring the kernels in the wash water for 1 min. After washing and draining, the nixtamal was ground in a stone mill (FUMASA, M100, Queretaro) into corn dough and then dried at room temperature with an electric fan until it reached a moisture content of 12% w/w (method 925.10) [13]. Then, the nixtamal flour was pulverized in a hammer mill (PULVEX 200, Mexico City, Mexico).

The nixtamal was hydrated to obtain the dough to prepare the tortillas with a manual press to obtain disks with a 20 cm of diameter and 1 mm of thickness. The disks were placed on a heating plate, and then were cooked for 1 min on each side at a temperature of approximately 250 °C. Subsequently, the tortillas were dried at room temperature to reach a moisture content of 12% *w/w*. The hardened tortillas were crushed and the particle size was homogenized in a sieve No. 4 (USA series) prior to grinding. On the other hand, a portion of crushed tortillas was ground in a hammer mill (PULVEX 200, Mexico City, Mexico) to obtain flour without storage for analysis (TF0). Another portion of crushed tortillas was stored at refrigeration temperature (4 °C) for 7, 15, and 30 days. Then, the tortillas were pulverized in a hammer mill (PULVEX 200, Mexico City, Mexico) to obtain samples TFR7, TFR15, and TFR 30, respectively.

The nixtamalized corn tortilla flour obtained by the industrial method, named as commercial flour (CF), was purchased from a local establishment considering a recent elaboration date. The dough was prepared according to the manufacturer’s instructions and the tortillas were made in a semi-industrial tortilla machine (Celorio, Model 70 KS). Subsequently, the tortillas were dried at room temperature to reach a moisture content of 12% *w/w*. The hardened tortillas were crushed and the particle size was homogenized in a sieve No. 4 (USA series) prior to grinding. As above-mentioned, a portion of crushed tortillas was ground in a hammer mill (PULVEX 200, Mexico City, Mexico) to obtain commercial flour without storage for analysis (CF0). As previously described, another portion of the crushed tortillas was stored at refrigeration temperature (4 °C) for 7, 15, and 30 days. Then, the tortillas were pulverized to obtain samples CFR7, CFR15, and CFR 30, respectively. 

### 2.2. Determination of the Power and Energy Consumption Average for Grinding

The hardened tortillas were triturated by using a hammer mill, described in the previous section, and a dispenser was used to feed the feedstock to the mill. The used rate was 32.48 kgh^−1^ ± 0.47, which was constant for all samples to produce a muffed grinding. A mesh of 0.8 mm was placed at the output restriction. The energy (E) and power (P) required for grinding were determined according to equations reported by Gutiérrez-Cortez et al. [14]. 

### 2.3. Texture Analysis of Hardened Tortillas

Hardened tortillas were analyzed at room temperature in a texture analyzer (Instron Texture Technologies Corp., Hamilton, MA, USA), according to the method reported by Ochoa-Martinez et al. [15].

### 2.4. Proximal Chemical Analysis

Flours obtained from the different treatments were subjected to a proximal chemical analysis in accordance with the methods established by the Association of Official Analytical Chemists (AOAC, 2000) [13] and the American Association of Cereal Chemists (AACC, 2000) [16]. The analysis included the determination of crude fiber (Method 962.09) [13], crude protein (Method 955.04) [13], moisture (Method 925.10) [13], lipids (Method 30–25) [16], ashes (Method 08-01) [16], and nitrogen free extract calculated by difference (%NFE = 100 − [%moisture + %Crude fiber + %Crude protein + %Ether extract + %Ash]).

American Association of Cereal Chemists

### 2.5. Resistant Starch Content Analysis

The analysis of resistant starch was carried out with a commercial kit (R-Star, Megazyme^®^, Bray, Ireland). The samples were subjected to protein hydrolysis with pepsin (3200–4500 U/mg, Sigma Chemical Co. St. Louis MO, USA) at acid medium (pH = 2.0) and incubated for 30 min at 37 °C in a shaking water bath to simulate stomach conditions, followed by hydrolysis of starch with pancreatic α-amylase (3 Ceralpha U/mg) for 16 h and pH close to neutrality. After centrifugation (1500 g for 10 min), the products of the hydrolysis were removed. The indigestible starch fraction remaining in the residue was dispersed in an alkaline medium (2 mL of 2 M KOH to each sample, J.T. Baker Center Valley, PA, USA) and then hydrolyzed with amyloglucosidase (300 U/mL). Subsequently, 8 mL of 1.2 M sodium acetate buffer (pH = 3.8, J.T. Baker Center Valley, PA, USA) was added to each sample with stirring. Immediately, 0.1 mL of amyloglucosidase (300 U/mL) were added to the samples and mixed. The samples were placed in a water bath at 50 °C and incubated for 30 min with intermittent mixing on a vortex mixer. Then, the samples were transferred to a 100 mL volumetric flask and adjusted to 100 mL with distilled water and mixed. A 10 mL aliquot of the solution was centrifuged at 1500 g for 10 min (Heraeus, Thermo Scientific, Bartlesville, OK, USA). Two aliquots of 0.1 mL were transferred into glass test tubes (16 × 100 mm) and 3.0 mL of glucose oxidase plus peroxidase and 4-aminoantipyrine (GOPOD) reagent (prepared according to the manufacturer’s instructions) were added to the test tubes. Successively, the tubes were incubated at 50 °C for 20 min. Finally, the absorbance of each solution was measured at 510 nm (VE-5100 UV, Velab, Pharr, TX, USA) against the reagent blank (0.1 mL of 100 mM sodium acetate buffer at pH = 4.5 and 3.0 mL of GOPOD reagent) and compared with the absorbance of D-glucose standards prepared with 0.1 mL of D-glucose (1 mg/mL) and 3 mL of GOPOD. 

### 2.6. Calcium Content Analysis

The calcium content in experimental samples was determined by the dry ashing method (968.08) [13]. In brief, 0.5 g of flour were placed in glazed silica crucibles and weighed to the nearest 0.0001 g. The samples were then placed in a muffle furnace maintained at 550 °C. After 2 h, the crucibles were removed, cooled, and the residue was treated with 3 M HCl (J.T. Baker Center Valley, PA, USA) to dissolve the residues. The content of each crucible was transferred to 100 mL volumetric flasks and diluted to volume with distilled water. The calcium concentration in the experimental samples was determined by atomic absorption spectrometry (Perkin Elmer, Mod. Analyst 300, Boston, MA, USA) with a flame detector. Calcium concentration in samples was determined with a standard calcium solution and a calibration curve. The operating conditions were similar to those reported by Gutiérrez et al. [12].

### 2.7. Color Measurement

The color of the flours was determined with a colorimeter (Minolta, CR300, Tokyo, Japan) using the granular solids device and expressed as Hunter L, a, and b color values. Color values were recorded as L, darkness/lightness (0, black; 100, white); a (−a, greenness; +a, redness); and b (−b, blueness; +b, yellowness). The color measurements were repeated from three different positions [17]. 

### 2.8. Particle Size Distribution

Particle size distributions of samples were measured by using a RO-TAP equipment with a set of meshes (U.S. standard Rot-tap model KH59986-60) with a horizontal and vertical automatic stirrer, mesh numbers: 20, 25, 30, 35, 40, 45, 50, 60, 70, 80, 100, and pan). The separating procedure was done according to the ASAE Standards [18], where 100 g of flour was separated during 12 min with rigorous series. Then, the fractions retained on each one of the different meshes were collected and weighed. The fractionation process was performed in triplicate [14]. 

### 2.9. Water Absorption Index (WAI) and Water Solubility Index (WSI)

The WAI and WSI of the samples were determined using the procedure reported by Anderson [19]. In brief, a 2.5 g sample of flour (<60 mesh) was suspended in 3 mL of water at 30 °C in a 50 mL tared centrifuge tube, stirred intermittently for 30 min and centrifuged at 3000 g for 10 min (Heraeus, Thermo Scientific, Bartlesville, OK, USA). The supernatant liquid was decanted carefully into a tared evaporating dish. The remaining gel was weighed and the WAI calculated from this weight. The WSI was determined with the amount of dried solids recovered by evaporating the supernatant from the water absorption test and the result was expressed as a percentage of dry solids in the 2.5 g of sample.

### 2.10. Determination of Apparent Viscosity Profile

In this analysis, the samples were adjusted to a moisture content of 12%. The viscosity profile was obtained using a rheometer (Anton Paar, model MCR 102. St Albans, UK) that was programmed under the following conditions: the initial temperature of the system was adjusted to 50 °C and maintained for one minute. Subsequently, the sample was heated for 5.3 min from 50 to 90 °C and then the temperature remained constant at 90 °C for 5.3 min. Finally, the samples were cooled at 50 °C during 5.3 min and kept at this temperature for 1 min. All tests were performed with constant agitation at a speed of 193 rpm [20].

### 2.11. X-Ray Diffraction Analysis

Prior to the analysis, the flours were defatted and subsequently passed through a No. 100 USA series screen. The pulverized material was densely packed on an aluminum support. The analysis was carried out with x-ray diffraction equipment (Rigaku Ultima IV diffractometer, Tokyo, Japan) and using a wavelength of ʎ = 1.5406 Å. The equipment was operated at 35 kV and 15 mA. The measurements were made from 10 to 70° on a 2θ scale with a step size of 0.02° [21]. Additionally, the total relative crystallinity was obtained from the x-ray diffraction data by measuring the ratio of the crystalline and total area in the diffractogram (see Equation (1)) [22].

(1) $$Total\;relative\;crystallinity\;\left(\%\right)=\frac{Total\;area-amorphous\;area}{Total\;area}\times 100\;\;\;\;\;\;\;$$

### 2.12. Morphological Study

The morphological study of the experimental samples was carried out by low vacuum scanning electron microscopy (LV-SEM) by using a JSM 5600LV (Tokyo, Japan) microscope with a resolution of 5 nm, adjusted with an x-ray spectrometer, with dispersion energy (Noran instrument, Mod. Voyager 4.2.3). Prior to the analysis, the samples were placed in an aluminum sample holder attached with carbon tape. The analyses were carried out under the following conditions: an electronic acceleration voltage of 20 kV, with a pressure in the range of 12–20 Pa in the sample chamber.

### 2.13. Differential Scanning Calorimetry (DSC) Thermal Analysis

The thermal properties of samples were studied with a differential scanning calorimeter (DSC1 model 821, Mettler Toledo, Greifensse, Switzerland) previously calibrated with indium. Gelation onset (To), peak (Tp), final (Tf) temperatures, and enthalpy change (ΔH) were obtained directly from the Mettler Toledo analysis software for Windows, according to Amador-Rodríguez et al. [23] and Santiago-Ramos et al. [24]. Prior to the analysis, the samples were sieved at U.S. mesh (250 μm), then 3 mg of sample was weighed into an aluminum pan and added to 7.5–8.5 mg of deionized water. The pan was sealed tightly, and the empty aluminum pan was used as a reference. The sample was heated at a temperature ranging from 30 to 130 °C with a heating rate of 10 °C/min. Each sample was run in triplicate.

### 2.14. Statistical Analysis

The results obtained from the physicochemical characterization of samples were analyzed with an analysis of variance (ANOVA) followed by a Tukey’s test with α = 0.05 and in all cases, the statistical package SPSS version 2.2 was used. At least three to five replicates for the measurements were carried out depending on the analysis and an average value is reported.

## 3. Results and Discussion

### 3.1. Power and Energy Consumption for Grinding

Table 1 shows the values observed for the hardness, flour yield, power, and energy consumed during the grinding of hardened tortillas obtained by the traditional and industrial method without storage and stored in refrigeration. It is evident that by increasing the refrigeration storage time in the tortillas made with the traditional and industrial (commercial) methods, the power, energy consumption, and the hardness decreased significantly (*p* ≤ 0.05) with values of 21.42, 40.75, and 17.42%, respectively in comparison with the tortilla flours without storage. However, the highest values were detected in tortillas made with the traditional method. The refrigeration storage time increases the tendency of tortillas to crack (friability) and consequently, require a lower energy input during milling. On the other hand, as the storage time in refrigeration is prolonged, the flours yield decreased (6.14%) with statistically significant differences (*p* ≤ 0.05) in comparison with tortilla flours without storage. It has been pointed out that the texture attributes of foods depend on the structure and composition that are obtained by submitting the ingredients to a sequence of operations, which comprise a given food process [25]. This means that during storage, the composition of the tortillas was modified, resulting in the transformation and destruction of the original structures in the food including the development of new structures, which is reflected in an increment in terms of weakness in the tortilla, thus a lower energy input is needed to cause fracturing. The hardness values observed in all experimental samples were higher than those reported by Ochoa-Martínez et al. [15] and Matiacevich et al. [26] for tortilla chips (0.8 to 2.5 kg_force_), which can be attributed to starch retrogradation during tortillas storage. Starch is the main carbohydrate constituent of tortillas, when starch is cooked in excess of water, as happens in the nixtamalization process, starch gelatinization takes place. Then, when starch pastes are stored for some time (hours or days), retrogradation (a recrystallization phenomenon) occurs, which is responsible of textural and starch digestibility changes during storage of starch-based products [9,27].

### 3.2. Chemical Composition of Traditional and Industrial Corn Tortilla Flours

The proximal chemical analyses of flour samples are summarized in Table 2. The moisture content in all experimental samples was in a range between 8.44 and 8.77%. These values were within the Mexican Standard for nixtamalized corn flours (NMX-F-046-S-1980) [28], which specifies a maximum value of 11%.

The protein and fat content in samples were slightly lower than the NMX-F046-S-1980 standard [28] (minimum values of 8 and 4% for protein and fat, respectively). Nevertheless, the protein content in commercial corn tortilla flours was significantly higher (*p* ≤ 0.05) than the values observed for traditional corn tortilla flours; whereas significant low values (*p* ≤ 0.05) for the fat content were observed in commercial corn tortilla flours in comparison with traditional corn tortilla flours. These results were similar with those reported by Bello-Perez et al. [29] in traditional and commercial tortillas for protein (7.82 and 7.73%, respectively) and fat (3.63 and 3.41%, respectively) contents. On the contrary, the protein and fat contents observed in this study exhibited higher values compared with contents detected in nixtamalized corn tortillas from different points of sale in Mexico City [30]. Crude fiber and ash contents were higher in traditional corn tortilla flours, although commercial corn tortilla flours passed the standard set by NMX-F046-S-1980 [28] for crude fiber content (maximum 2%). The ash contents of traditional and commercial corn tortilla flours were more than the NMX-F046-S-1980 [28] standard (maximum 1.5 %). It is important to note that ash contents in the experimental samples were different to those reported previously for traditional and commercial corn tortillas [29,30]. Regarding to calcium content, no significant differences (*p* ≤ 0.05) were detected in the content of this mineral in the experimental samples due to refrigeration storage. Nevertheless, it is evident that the calcium content in traditional corn tortilla flours was significantly higher (approximately 133 %) when compared to commercial corn tortilla flours. These data were in accordance with the calcium content reported previously [30,31,32] for instant corn flours and tortillas prepared with traditional and industrial methods. Undoubtedly, differences in the chemical composition of the experimental samples can be attributed to different factors such as maize phenotype, grain quality (fractured grain percentage), and mainly to the nixtamalization method used to prepare the tortillas [30]. Concerning this, the lime amount added in the traditional method is between 1 to 2% *w/w* of the corn grain; while in the industrial method, it varies from 5 to 6% *w/w* of corn grain. Additionally, the traditional nixtamalization process involves longer steeping times than the industrial process, which facilitates water and calcium diffusion into the anatomical structures of corn grain, allowing the starch gelatinization and increasing the mineral content [12,31]. In several industrial nixtamalization processes, the hull or pericarp (bran) of corn kernel removal during the washing step is common practice to avoid undesirable color sources to the nixtamalized corn products [33]. Additionally, an excessive washing of nixtamal is directly associated with dry matter loss, which implies the loss of fat, fiber, and minerals mainly located in the germ and pericarp [34]. This may explain lower contents of fat, crude fiber, ash, and calcium in industrial corn tortilla flours (22.08, 29.01 and 58.65%, respectively) than in traditional corn tortilla flours. 

**Figure 1:**
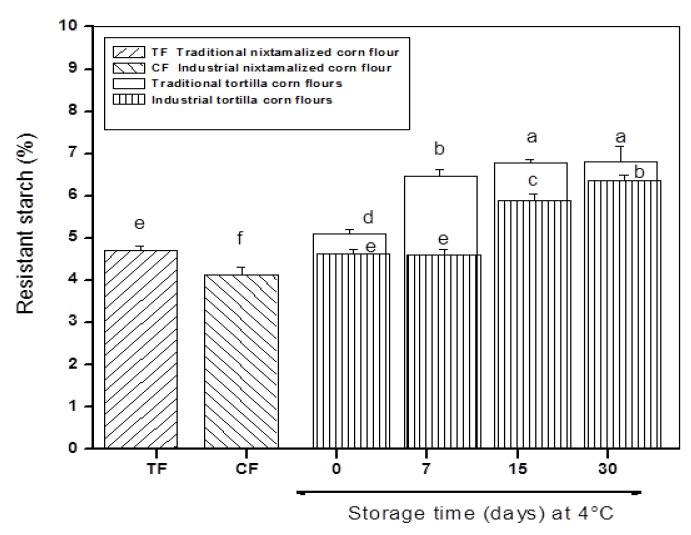
Resistant starch contents in traditional and commercial corn flours and corn tortilla flours. The values represent the mean ± standard deviation (SD), *n* = 5. Means in bars with different letters differ significantly (*p* ≤ 0.05).

The resistant starch (RS) content in traditional nixtamalized corn flour and traditional corn tortilla flours with and without refrigeration storage were significantly (*p* < 0.05) higher than the values observed in commercial nixtamalized corn flour and commercial corn tortilla flours (see Figure 1). It was observed that the increase of RS was 8.49 and 11.6%, respectively, in the transformation of flours into tortillas obtained with both traditional and industrial (commercial) methods. Santiago-Ramos et al. [24] described the increase in RS content from nixtamalized corn flour to tortilla using the traditional method. Nevertheless, increases detected in this study differed from values observed by those authors (≈50%), who mentioned that there are three types of RS in tortillas: RS5 (V-amylose-lipid complexes), RS3 (retrograded starch), and physically inaccessible native starch (RS1), and discrepancies can be attributed to maize variety and type of calcium salts used during thermo-alkaline treatment. 

It is important to note that RS content in samples increased as a function of refrigeration storage time, where the maximum values were detected at long storage times with an average relative resistant starch increase of 33.10 and 37.66% for traditional and commercial corn tortilla flours, respectively, in comparison to the unrefrigerated samples. The same trend was observed in previous reports, which mentioned that the starch retrogradation phenomenon is responsible for RS content rising [9,29,35,36]. However, the RS content reported by these authors exhibited lower RS values in tortillas stored at 4 °C for similar periods when compared with the results obtained in this study. Moreover, the RS content detected in the corn tortilla flours was greater than the values reported by Enriquez-Castro et al. [37] in extruded snacks, where the RS content generated during extrusion was very low (1.01%). At this point, it is important to empathize that drying and grinding of tortillas were additional steps to obtain corn tortilla flours, then these additional unitary operations exposed remaining native corn starch to gelatinization, increasing starch retrogradation. On the other hand, lower RS content in commercial corn tortilla flours can be related to the presence of hydrocolloids and gums added to commercial nixtamalized corn flours. It is well known that these compounds are added in tortilla processing to enhance the textural properties of tortillas (flexibility and strength) and reduce stickiness during processing and packaging [38,39]. However, it has been reported that hydrocolloids retard the starch retrogradation in refrigerated tortillas as these compounds avoid interactions between starch chains solubilized during gelatinization and consequently, RS content diminishes. In addition, it has been reported that the resistant starch content has a negative correlation with the hardness [40], which agrees with the data observed in this study (see Table 1), where corn tortillas that showed less resistance to fracture showcased the highest resistant starch content in flours.

### 3.3. Physicochemical Properties of Traditional and Commercial Tortilla Corn Flours 

Degree of lightness, which is associated with the *L* values, was significantly different (*p* ≤ 0.05) in corn tortilla flours prepared with different methods. This means that corn tortilla flours obtained by the industrial method (commercial flours) had the lightest color, while the corn tortilla flours obtained by the traditional method showed the least light color; therefore these flours were darker when compared to commercial tortilla corn flours (see Table 3). The color exhibited by the flours was yellow and the degree of yellowish color was light. This was supported by very low *a* values (−1 to 1 units) and minor *b* values (9–16 units). 

The darker color of the traditional corn tortilla flours is attributed to bran, which means that the more bran is removed, the lighter the flour produced [17]. Furthermore, the flour color has been related to the ash content, which is an indicator of bran and germ contamination in milling [41]. In this regard, the removal of corn pericarp and germ during nixtamalization denotes the loss of fiber and calcium [21,31]. This is supported by the lower content of crude fiber, ash, and calcium in corn tortilla flours obtained by the industrial method with respect to the tortilla flours prepared with the traditional method. Additional heat treatment to cook tortillas is responsible for lower *L* values in corn tortilla flours in comparison to NF and CF due to Maillard reactions, which are accelerated at high temperatures and pH [42].

**Figure 2:**
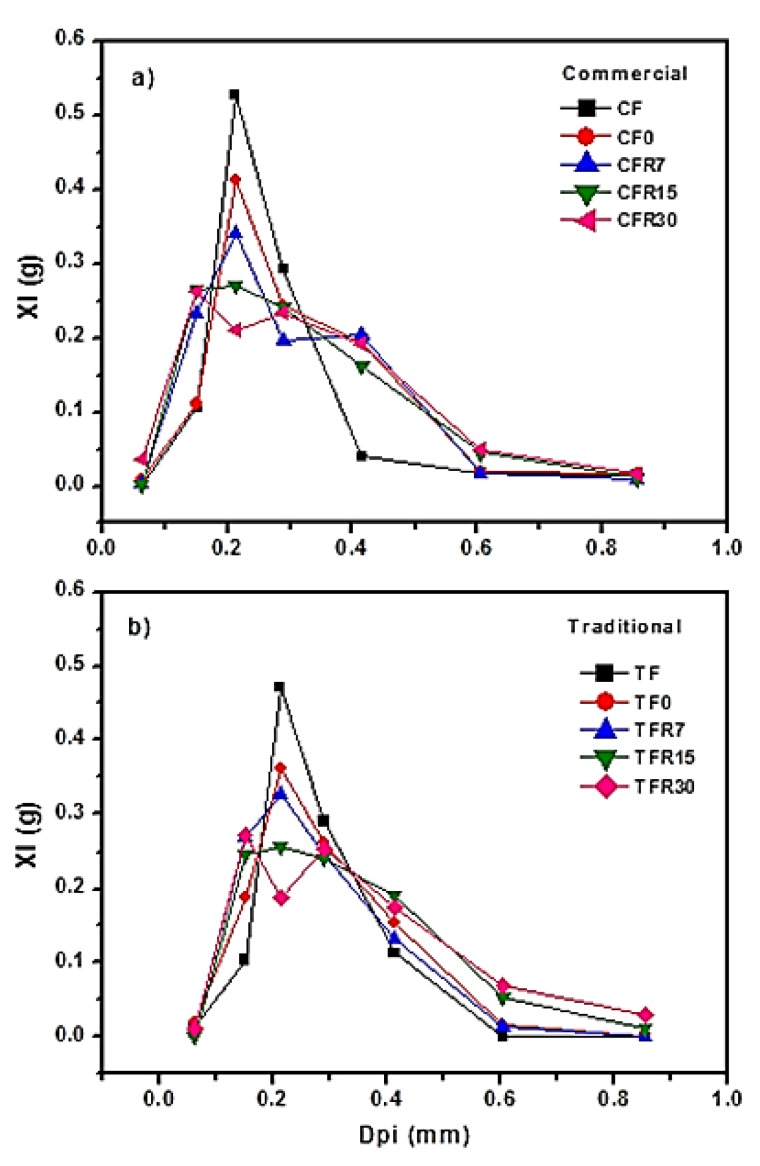
Particle size distribution of (**a**) nixtamalized corn flour and corn tortilla flours obtained by the industrial (commercial) method, (**b**) nixtamalized corn flour and corn tortilla flours obtained by de traditional method without refrigeration and stored in refrigeration (4 °C) at different periods of time. TF = Nixtamal flour made with the traditional method, CF = Nixtamalized corn flour made with the industrial (commercial) method, TF0 = traditional corn tortilla flour without refrigerated storage, CF0 = commercial corn tortilla flour without refrigerated storage, TFR(7, 15, 30) = traditional corn tortilla flours with refrigerated storage (4 °C) during 7, 15, and 30 days, respectively, CFR(7, 15, 30) = commercial corn tortilla flours with refrigerated storage (4 °C) during 7, 15, and 30 days, respectively.

The particle size distribution of the samples is shown in Figure 2. Figure 2a,b belong to the granulometric analysis of flours from corn tortillas nixtamalized by the commercial and traditional methods, respectively. The CF and TF samples showed greater homogeneity in terms of particle size with respect to the rest of the experimental samples, where the highest percentage of the material was retained in the sieve with an opening of 0.25 mm. The particle size distribution in these samples was very similar to those previously reported for corn flours nixtamalized by the industrial and traditional method [12,32]; however, the percentages of particle retained in this mesh were below the Mexican Standard for nixtamalized corn flours (NMX-F-046-S-1980) [28], which establishes a minimum value of 75%.

It should be noted that the samples stored in refrigeration, the particle size distribution shifted toward a greater content of coarse particles as the storage time increased as a bimodal particle size distribution was detected in the TFR30, CFR7, and CFR30 samples. This behavior was also observed in corn ground and distillers dried grains with solubles. It has been pointed out that there are several factors to consider that determine the particle size distribution and these are responsible for variations in this parameter, for example, mechanical, thermal, chemical, and biological stresses and shock on the original corn particles. Therefore, all of them contribute to the agglomeration of particles promoting particle size breakage, clumping, regrouping, and therefore redistribution, so that the net effect leads to larger particle size [43]. Particle size distribution is a fundamental criterion for nixtamalized corn flours, so that large particles are required for the textural characteristics of fried products (crispiness) as coarse particles disrupt the dough network, reduce blistering, and decrease oil uptake during frying. In contrast, small particles govern water absorption, viscosity, cohesiveness, plasticity, and smoothness [44]. In addition, it has been reported that extrudates produced with corn meal of high particle sizes expanded more than extrudates produced with small particle sizes [45]. This means that corn tortilla flours may be used as an ingredient to promote crispiness and to improve expansion in extruded products.

In nixtamalized corn flours, WAI is related to the starch crystalline structure disruption during thermo-alkaline treatment, so this parameter can be used as an index of gelatinization, since disrupted starch granules bind more water. Although WSI determines the amount of polysaccharides (i.e., dextrins) released from the granule that are water soluble, great values of WAI and WSI imply high starch granule fragmentation and dextrinization [46,47,48]. Significant differences *(p* < 0.05) were detected in the WAI values between traditional and commercial nixtamalized corn flours (TF and CF, respectively) as well as in traditional and commercial corn tortilla flours, where the highest values belonged to commercial corn tortilla flours (see Table 4). These results agreed with Campus-Baypoli et al. [49]. These authors observed an increase of WAI in the transformation of corn dough to tortilla and attributed higher WAI values in tortillas to the loss of the starch granules’ structure and integrity, which leads to starch gelatinization. Likewise, high values in WAI observed in commercial flours can also be explained by the presence of hydrocolloids, where hydroxyl groups in their structure allow for water interactions through hydrogen bonding [39].

Interestingly, it was observed that as the storage time at refrigeration increased, WAI and WSI diminished significantly (*p* ≤ 0.05) with a relative decrease of 10.30 and 4.90%, respectively, in traditional corn tortilla flours as well as 6.99 and 14.72%, respectively, in commercial corn tortilla flours. Neder-Suárez et al. [50] reported a similar trend in cornstarch extrudates stored at 4 °C. This may be explained by the formation of resistant starch (see Figure 1) due to re-association of amylose and amylopectin chains, which facilitates recrystallization (retrogradation) and promotes the formation of new compact molecular structures stabilized by hydrogen bonds, while avoiding the interaction of hydroxyl groups with water [51,52].

**Figure 3:**
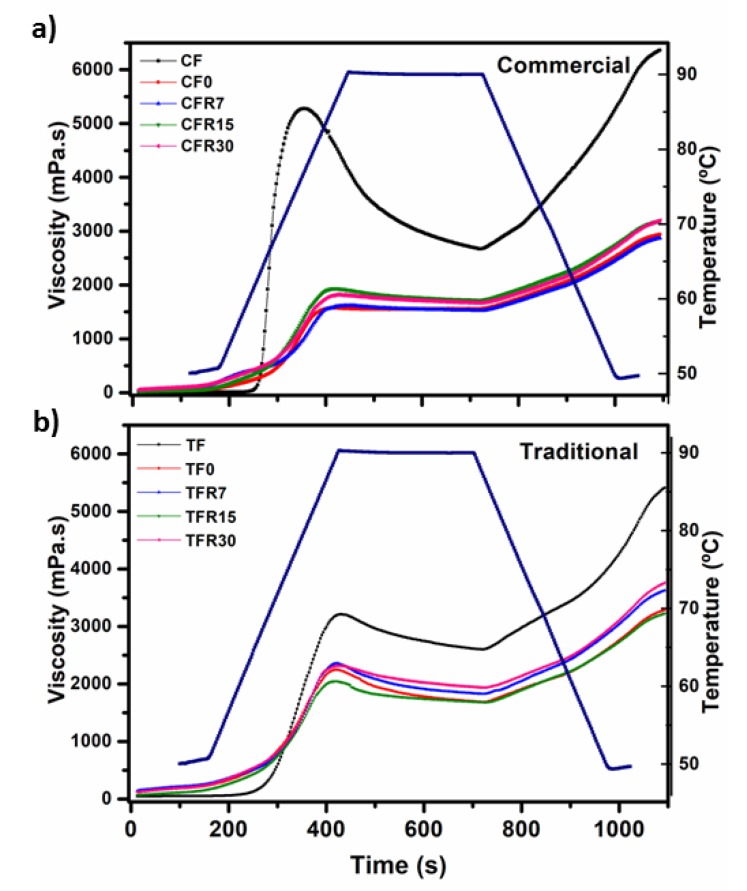
Pasting profiles of nixtamalized corn flours and corn tortilla flours obtained by the (**a**) industrial (commercial) and (**b**) traditional methods without refrigeration (TF, CF, TR0, CF0) and stored in refrigeration (4 °C) at different periods of time (7, 15, and 30 days).

The pasting profile of the experimental samples as a function of time is shown in Figure 3. It is evident that CF exhibited a higher peak viscosity (5305.76 mPa.s) in comparison to that observed in TF (3243.39 mPa.s). The highest peak viscosity in commercial flour may be due to the presence of hydrocolloids such as xanthan, guar, arabic, etc., as was previously mentioned, in order to improve the texture properties of the dough and tortilla, thereby favoring moisture retention and therefore making the tortilla harden more slowly [39,53,54]. Regarding this, Calderón-Peralta et al. [55] reported that the nixtamalized corn flours with the addition of xanthan gum and *Hymenaea courbaril* gum peak viscosity values were higher than those for the control. On the contrary, the peak viscosity in the commercial corn tortilla flours was lower than the peak viscosity in CF. The peak viscosity in the traditional corn tortilla flours was significantly higher (*p* ≤ 0.05) with respect to the peak viscosity observed in the commercial corn tortilla flours, whose averages were 2302.94 ± 216.99 and 1789.16 ± 119.16 mPa*s, respectively (see Figure 3a,b). This means that in commercial corn tortilla flours, fewer starch granules are susceptible to gelatinization.

CF displayed a noticeable breakdown during the holding period at 90 °C, while TF showed a slight breakdown and CF showed a higher setback upon cooling in comparison to TF. On the other hand, traditional corn tortilla flours presented higher setback upon cooling than that exhibited by commercial corn tortilla flours. This behavior has been related to a structural rearrangement and associations of starch chains as well as changes of crystalline order in corn starch due to the nixtamalization process [32,56,57]. The highest peak viscosity values in traditional corn tortilla flours can be explained from two points of view: (1) the presence of ungelatinized starch, taking into account that during the traditional nixtamalization process, a partial starch gelatinization takes place (proximately 20% of starch) [58] and (2) the existence of negatively charged gums in commercial corn tortilla flours, for example, carboxymethyl cellulose and xanthan gum as the polymers in starch suspensions delay granule pasting/destruction and amylose leaching, thus delaying pasting and reducing the peak viscosity [59]. Thus, the peak viscosity in CF is mainly attributed to gums. Regarding this, it is important to mention that the viscosity of the gums is dependent on time, temperature, particle size, concentration, and other factors. Therefore, it is possible that during the milling and storage of the tortillas, the functional properties of the gums were modified [60].

**Figure 4:**
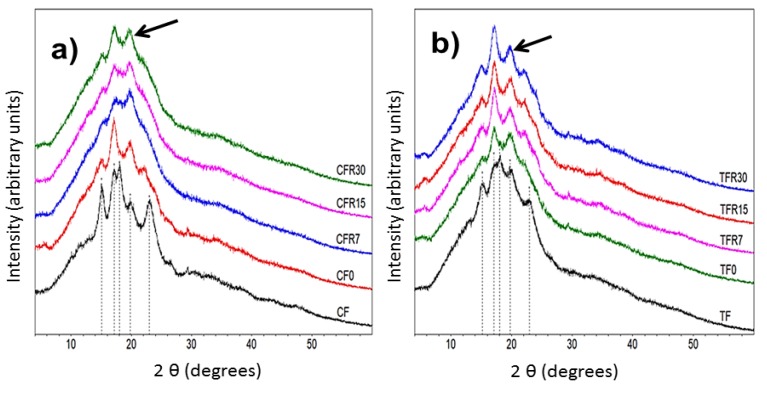
X-ray diffraction pattern of corn flours and corn tortilla flours obtained by the (**a**) industrial (commercial) and (**b**) traditional methods without refrigeration (TF, CF, TR0, CF0) and stored in refrigeration (4 °C) at different periods of time (7, 15, and 30 days).

Figure 4 shows the x-ray diffraction patterns of experimental samples. The CF displayed an A-type x-ray diffraction typical for cereal starches with defined peaks at 2*θ* angles of 15, 17, 18, and 23° and similar to those reported by Rojas-Molina et al. [58] and Sullivan et al. [61] for corn and wheat native starches (Figure 4a). The TF exhibited a similar x-ray diffraction pattern observed in CF, however, with undefined peaks. This indicates more pronounced damage in the crystalline structure of native starch in the traditional nixtamalization process when compared with the industrial method (Figure 4b). In the corn tortilla flours, an increase in the relative intensity of the peak at a 2*θ* angle of ≈20°, accompanied by a better definition of it, was detected in both methods (traditional and industrial) (see arrows in Figure 4a,b). There was also a loss in definition of the peaks located at 15, 17, and 23° in the 2*θ* scale when the refrigeration storage time was extended, these data are associated with the total relative crystallinities in the samples, which are discussed below. The peak located at ≈20° belongs to the formation of a Type V amylose–lipid complex (resistant starch type 5 or RS5), which has been identified in tortillas from an ecological nixtamalization process, tamales and corn snacks [7,24,62], and consequently, this is reflected in an increase in the resistant starch content (see Figure 1). The loss in definition of the peak at 15° from the transformation of corn flour to tortilla has been associated with two phenomena: (1) modifications of side branches of amylopectin to form amylose helices that lead to an increase of the amylose–lipid complex peak at 20° and (2) type of lipid, where the higher the peak, the longer the fatty acid chain [63,64]. 

**Figure 5:**
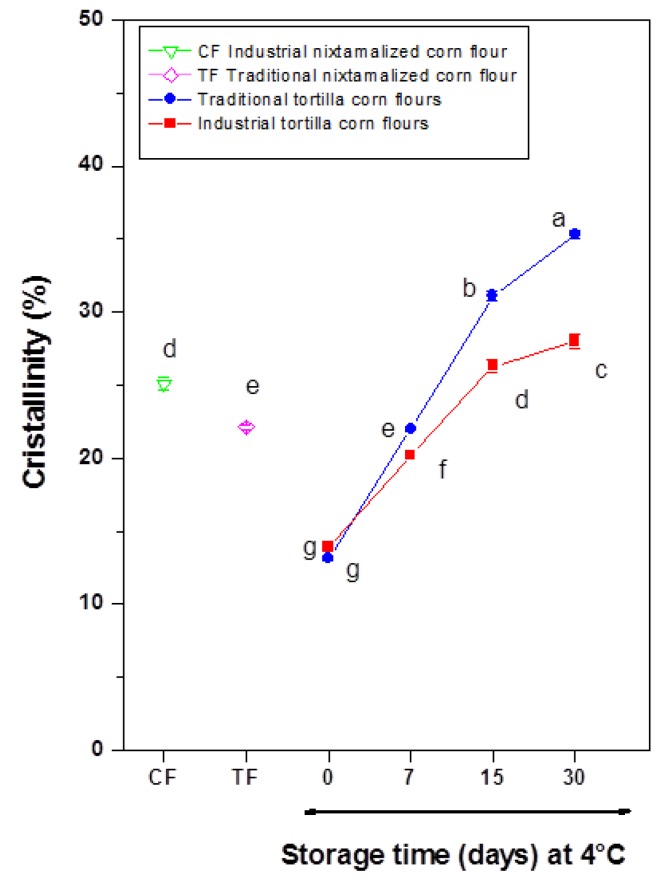
Total relative crystallinities of corn flours and corn tortilla flours obtained by the industrial (commercial) and traditional methods without refrigeration (TF, CF, TR0, CF0) and stored in refrigeration (4 °C) at different periods of time (7, 15, and 30 days). Different letters means differ significantly.

Total relative crystallinities of experimental samples are shown in Figure 5. Crystallinity in CF (25.1 ± 0.46%) was significantly higher (*p* ≤ 0.05) than that observed in TF (22.1 ± 0.15%). The transformation of CF and TF into tortillas decreased the crystallinity between 45 and 50%, respectively. Similarly, Santiago-Ramos et al. [65] reported that nixtamal starch of intermediate and soft grains showed a decrease in crystallinity (≈32%) when it is transformed into tortilla due to the partial gelatinization that occurs during cooking. It is evident that there was a statistically significant increase (*p* ≤ 0.05) in crystallinity when refrigeration storage time was extended in traditional corn tortilla flours (≈168%) and industrial corn tortilla flours (≈101%). This increase can be explained by the amylose–lipid complex Type V formation (see Figure 4). Our data agrees with Sullivan et al. [61], since they reported a sharp increase in crystallinity of baker’s flour stored after eight days at 3.5 °C, which is attributable to the RS formation. 

**Figure 6:**
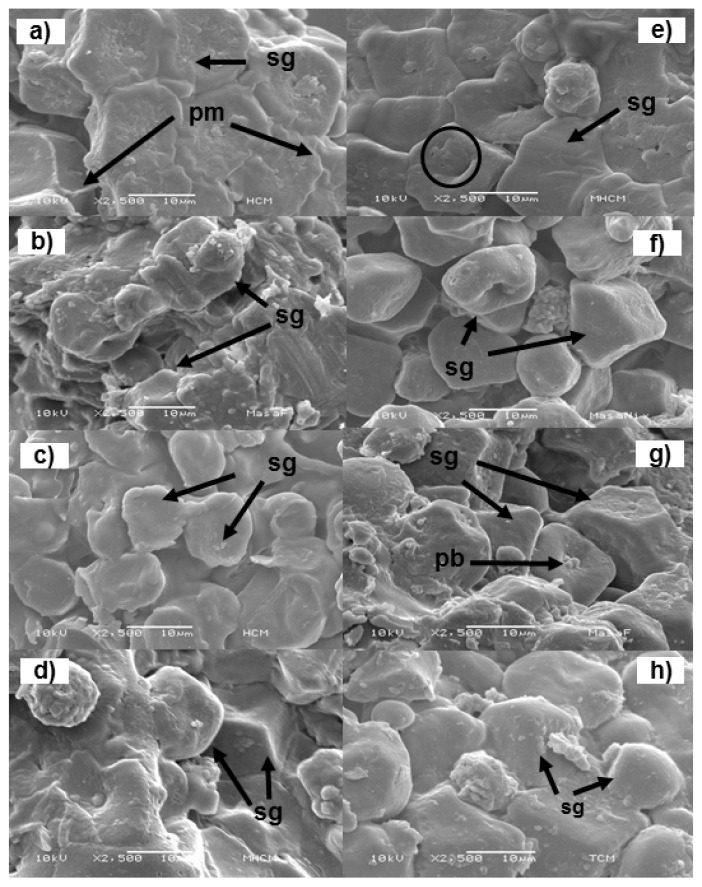
Scanning electron microscope (SEM) images of nixtamalized corn flours and corn tortilla flours obtained by the industrial (**a**–**d**) and traditional methods (**e**–**h**). (**a**) Commercial nixtamalized corn flour, (**b**) commercial corn tortilla flour without refrigeration, (**c**) commercial corn tortilla flour stored at 4 °C for 7 days, (**d**) commercial corn tortilla flour stored at 4 °C for 30 days, (**e**) traditional nixtamalized corn flour, (**f**) traditional corn tortilla flour without refrigeration, (**g**) traditional corn tortilla flour stored at 4 °C for 7 days, (**h**) traditional corn tortilla flour stored at 4 °C for 30 days. sg = starch granules, pm = protein matrix, pb = protein bodies.

Scanning electron microscopy (SEM) images of corn flours and corn tortilla flours are shown in Figure 6. Commercial nixtamalized corn flour showed granules with a regular polygonal shape with no damage and covered with the protein matrix (Figure 6a). The traditional nixtamalized corn flour exhibited starch granules with a polygonal shape (Figure 6e); nevertheless, some granules showed cavities (see black circle) and the protein matrix was scarcely noticeable and less rough, which implies a partial solubilization [66]. The morphological modifications were related with starch damage during the nixtamalization process, as has been previously reported [12]. Most likely, the presence of gums in commercial nixtamalized corn flours helps starch granules to retain their polyhedral morphology as described by Calderón-Peralta et al. [55]. Figure 6b,f show the commercial and traditional corn tortilla flours without refrigeration, respectively, where a gradual disaggregation of starch granules is evident due to the milling process. Nevertheless, traditional corn tortilla flour exhibited starch granules of larger size or collapsed (see arrow) and with a greater number of gaps between them (Figure 6f) in comparison to the commercial corn tortilla flour (Figure 6b). In both samples, the gradual loss of the polyhedral form of the starch granules was clearly visible, which implies a partial starch gelatinization. The grinding of tortillas favored the gelatinization of the whole starch granules as milling is a thermomechanical process, which promotes 15% of the gelatinization during the transformation of corn into tortillas [67].

Figure 6c,g show commercial and traditional corn tortilla flours refrigerated at 4 °C for seven days, respectively. Figure 6c shows starch granules with a smooth surface paste, while Figure 6g shows dispersed starch granules embedded into the protein matrix (pm) and protein bodies (pb) on the surface. It is clear that the size of the starch granules and the space between them were larger with respect to those observed in Figure 6c. Regarding this, it has been reported that hydrocolloids delay tortilla retrogradation due to less starch gelatinization, avoiding the leaching of amylose during tortilla processing and storage [55]. This explains the lower values in peak viscosity and resistant starch content in commercial corn tortilla flours than those detected in traditional corn tortilla flours (see Figure 1 and Figure 3). Figure 6d,h belong to commercial and traditional corn tortilla flours refrigerated at 4 °C for 30 days, respectively. Both micrographs images show a different morphology in comparison to previous images, where angular shaped and partially damaged starch granules remain embedded in a matrix. It is most likely that leached amylose chains from starch granules and subsequent retrogradation are responsible for this new microstructural order [68].

Table 5 shows the thermal properties of nixtamalized corn flours and corn tortilla flours with and without refrigeration. In all samples, three endothermic events were found. The first one belongs to the corn starch gelatinization. There were significant differences (*p* < 0.05) in T_o_, T_p_, T_f_, and ΔH of flours obtained by different nixtamalization methods. The T_o_ (70.23 °C), T_p_ (78.15 °C), and T_f_ (80.17 °C) of TF were similar to values reported by Liu, Yuan, and Wang [66] for dry milling corn flour. On the other hand, the T_o_ (65.19 °C), T_p_ (67.30 °C), and T_f_ (69.28 °C) of CF were significantly lower (*p* < 0.05) than TF, which can be attributed to the granule size and distribution of starch [69]. Gelatinization enthalpy of CF (4.04 J/g) was significantly lower (*p* < 0.05) than that detected in TF (5.30 J/g). These results coincide with those reported by Calderón-Peralta et al. [55] and Aguirre-Cruz et al. [70], who mentioned that ΔH values diminish due to hydrocolloid–starch interaction (which retains more water molecules) promoting higher mobility during heating, increasing kinetic energy, and decreasing the enthalpy values. In general, both traditional and commercial corn tortilla flours showed lower transition temperatures and enthalpy values as storage time increased. This may be explained by the retrogradation phenomenon as retrograded starches show lower enthalpies and transition temperatures than native starches because they have weaker starch crystallinity [71]. 

The second and third endotherms in the samples were detected with two peak temperatures: the first one in a range from 100.2 to 116.04 °C and the second one between 107.18 and 135.91 °C, respectively. These transition temperatures have been associated with the melting of the amylose–lipid complexes Types I and II, respectively. Nevertheless, it is important to denote that in TF, CF and corn tortilla flours obtained with the traditional and commercial nixtamalization methods without storage, the transition temperatures and enthalpy values for lipid complexes Types I and II agreed with the values reported previously [65]. Nonetheless, the To_RS5I_**,** To_RS5II_, ΔH_RS5I_, and ΔH_RS5II_ values in the samples increased as a function of storage time. Enthalpy changes on the dissociation of amylose–lipid complexes involve the breaking of intrahelical hydrogen-bonds (Type I) and interhelical hydrogen bonds (Type II) [72]. It is likely that these bonds get stronger with storage time. 

## 4. Conclusions

The search for simple technologies for increasing the content of nutraceutical ingredients in foods and reduce the waste of raw materials is a challenge for the food industry. 

In this study, the physicochemical properties and resistant starch content of corn tortilla flours refrigerated at different storage times were investigated. The results showed that the storage time in refrigeration promoted the friability of the dehydrated tortillas and therefore, less energy was required during milling to obtain corn tortilla flours. However, the particle size distribution of these flours allowed for a higher percentage of coarse particles to be obtained. The nixtamalization method (traditional and industrial) to prepare tortillas modified the nutritional composition of the corn tortilla flours, mainly in resistant starch content, which was significantly higher in the corn tortilla flours obtained by the traditional method, which increased with the storage time in refrigeration. This means that during tortilla milling and subsequent storage in refrigeration, gelatinization and retrogradation of the remaining native starch took place, which entailed the formation of lipid–starch complexes (resistant starch Type V).

The WAI and WAS values in the commercial corn tortilla flours were higher than in the traditional corn tortilla flours. Nevertheless, the apparent viscosity, temperature, and enthalpy of starch gelatinization were lower than those detected in traditional corn flours.

The physicochemical characteristics and the resistant starch content of the corn tortilla flours show their potential to be used as nutraceutical ingredients for future product development. However, further studies are needed to determine physicochemical, sensory, quality attributes, and relationships between such characteristics and the formulation of products with added corn tortilla flours. 

## Figures and Tables

**Table 1:** Hardness, flour yield, power and energy consumption during grinding of corn tortillas obtained by the traditional and industrial (commercial) methods.

Sample	Power (W)	Energy (W.h/kg)	Hardness Kg_force_	Flour Yield (g)
TF	1031.86 ± 27.54 ^f^	15.78 ± 2.21 ^e^	NE	58.76 ± 1.39 ^c^
CF	NE	NE	NE	66.04 ± 1.62 ^a^
TF0	1512.58 ± 15.19 ^a^	36.58 ± 3.19 ^a^	4.73± 0.47 ^a^	56.59 ± 0.15 ^cd^
CF0	1441.96 ± 27.48 ^b^	33.40 ± 2.74 ^b^	3.66 ± 0.15 ^c^	54.49 ± 0.98 ^d^
TFR7	1402.59 ± 24.05 ^c^	33.78 ±2.15 ^ab^	4.04 ± 0.71 ^b^	61.74 ± 0.79 ^b^
CFR7	1382.67 ± 17.60 ^c^	29.17 ± 1.51 ^c^	3.33 ± 0.07 ^d^	56.63 ± 0.95 ^cd^
TFR15	1205.08 ± 25.35 ^d^	23.32 ± 0.02 ^d^	3.87 ± 0.21 ^bc^	50.94 ± 1.018 ^e^
CFR15	1115.03 ± 18.79 ^e^	17.67 ± 1.71 ^e^	3.23 ± 0.06 ^d^	51.76 ± 1.59 ^e^
TFR30	931.15 ± 16.01 ^g^	10.54 ± 0.97 ^f^	3.21 ± 0.14 ^d^	45.35 ± 1.67 ^f^
CFR30	928.11 ±19.55 ^g^	9.87 ± 0.30 ^f^	3.06 ± 0.06 ^d^	46.41 ± 1.40 ^f^

The values represent the mean ± standard deviation (SD), *n* = 5. Means in columns with different letters differ significantly (*p* ≤ 0.05). NE = Not evaluated. TF = Nixtamal flour made with the traditional method, CF = Nixtamalized corn flour made with the industrial (commercial) method, TF0 = traditional tortilla corn flour without refrigerated storage, CF0 = commercial tortilla corn flour without refrigerated storage, TFR(7, 15, 30) = traditional tortilla corn flours with refrigerated storage during 7, 15, and 30 days, respectively, CFR(7, 15, 30) = commercial tortilla corn flours with refrigerated storage during 7, 15, and 30 days, respectively.

**Table 2:** Chemical proximate analysis and calcium content of corn flours and corn tortilla flours (g/100 g).

Samples.	Moisture	Protein	Fat	Crude Fiber	Ashes	NFE	Calcium
**TF**	8.77 ± 0.05 ^a^	7.40 ± 0.10 ^bcd^	4.10 ± 0.05 ^a^	2.60 ± 0.10 ^a^	2.32 ± 0.10 ^a^	74.81	0.21 ± 0.04 ^a^
**CF**	8.53 ± 0.05 ^bc^	7.70 ± 0.10 ^a^	3.12 ± 0.07 ^b^	1.86 ± 0.15 ^b^	1.51 ± 0.12 ^b^	77.28	0.08 ± 0.02 ^b^
**TF0**	8.64 ± 0.10 ^ab^	7.36 ± 0.05 ^cd^	3.97 ± 0.11 ^a^	2.65 ± 0.10 ^a^	2.42 ± 0.17 ^a^	74.96	0.23 ± 0.03 ^a^
**CF0**	8.64 ± 0.12 ^ab^	7.64 ± 0.20 ^a^	3.17 ± 0.07 ^b^	1.86 ± 0.15 ^b^	1.61 ± 0.11 ^b^	77.08	0.10 ± 0.01 ^b^
**TFR7**	8.58 ± 0.01 ^abc^	7.16 ± 0.25 ^d^	3.92 ± 0.11 ^a^	2.53 ± 0.20 ^a^	2.32 ± 0.10 ^a^	75.49	0.19 ± 0.01 ^a^
**CFR7**	8.54 ± 0.08 ^bc^	7.60 ± 0.10 ^abc^	3.09 ± 0.07 ^b^	1.96 ± 0.15 ^b^	1.61 ± 0.12 ^b^	77.20	0.09 ± 0.01 ^b^
**TFR15**	8.44 ± 0.22 ^c^	7.30 ± 0.10 ^d^	4.20 ± 0.20 ^a^	2.63 ± 0.10 ^a^	2.25 ± 0.05 ^a^	75.18	0.20 ± 0.01 ^a^
**CFR15**	8.57 ± 0.11 ^bc^	7.63 ± 0.06 ^ab^	3.13 ± 0.22 ^b^	1.83 ± 0.25 ^b^	1.64 ± 0.13 ^b^	77.2	0.08 ± 0.01 ^b^
**TFR30**	8.66 ± 0.10 ^ab^	7.33 ± 0.21 ^d^	3.95 ± 0.07 ^a^	2.70 ± 0.17 ^a^	2.28 ± 0.03 ^a^	75.08	0.21 ± 0.02 ^a^
**CFR30**	8.70 ± 0.10 ^ab^	7.66 ± 0.15 ^a^	3.20 ± 0.13 ^b^	1.80 ± 0.17 ^b^	1.67 ± 0.07 ^b^	76.97	0.08 ± 0.02 ^b^

The values represent the mean ± standard deviation (SD), *n* = 5. Means in columns with different letters differ significantly (*p* ≤ 0.05). NFE = Nitrogen free extract. TF = Nixtamal flour made with the traditional method, CF = Nixtamalized corn flour made with the industrial (commercial) method, TF0 = traditional tortilla corn flour without refrigerated storage, CF0 = commercial tortilla corn flour without refrigerated storage, TFR(7, 15, 30) = traditional tortilla corn flours with refrigerated storage during 7, 15, and 30 days, respectively, CFR(7, 15, 30) = commercial tortilla corn flours with refrigerated storage during 7, 15, and 30 days, respectively.

**Table 3:** Color of traditional and industrial (commercial) nixtamalized corn flours and corn tortilla flours.

Sample	L	a	b
**TF**	64.01 ± 0.16 ^b^	0.10 ± 0.01 ^c^	9.04 ± 0.05 ^d^
**CF**	68.86 ± 0.46 ^a^	−1.04 ± 0.02 ^d^	10.73 ± 0.07 ^d^
**TFR0**	55.62 ± 0.18 ^c^	0.31 ± 0.01 ^b^	12.12 ± 0.03 ^c^
**CFR0**	64.60 ± 1.06 ^b^	0.35 ± 0.03 ^a^	13.85 ± 0.08 ^b^
**TFR7**	56.42 ± 0.65 ^c^	0.32 ± 0.03 ^b^	13.75 ± 0.11 ^b^
**CFR7**	66.74 ± 0.18 ^b^	0.35 ± 0.05 ^a^	14.21 ± 0.04 ^b^
**TFR15**	54.04 ± 0.55 ^c^	0.30 ± 0.03 ^b^	14.43 ± 0.06 ^b^
**CFR15**	65.24 ± 0.11 ^b^	0.36 ± 0.02 ^a^	14.89 ± 0.06 ^b^
**TFR30**	55.39 ± 0.15 ^c^	0.31 ± 0.01 ^b^	15.42 ± 0.09 ^a^
**CFR30**	66.66 ± 0.12 ^b^	0.36 ± 0.02 ^a^	16.25 ± 0.11 ^a^

The values represent the mean ± standard deviation (SD), *n* = 3. Means in columns with different letters differ significantly (*p* ≤ 0.05). TF = Nixtamal flour made with the traditional method, CF = Nixtamalized corn flour made with the industrial (commercial) method, TF0 = traditional corn tortilla flour without refrigerated storage, CF0 = commercial corn tortilla flour without refrigerated storage, TFR(7, 15, 30) = traditional corn tortilla flours with refrigerated storage (4 °C) during 7, 15, and 30 days, respectively, CFR(7, 15, 30) = commercial corn tortilla flours with refrigerated storage (4 °C) during 7, 15, and 30 days, respectively.

**Table 4:** Water absorption index (WAI) and water solubility index (WSI) of nixtamalized corn flours and corn tortilla flours obtained by the traditional and industrial methods.

Sample	WAI (%)	WSI (%)
**TF**	3.71 ± 0.17 ^d^	4.69 ± 0.20 ^a^
**CF**	4.86 ± 0.15 ^c^	4.62 ± 0.17 ^ab^
**TF0**	5.26 ± 0.22 ^b^	3.71 ± 0.15 ^bc^
**CF0**	5.72 ± 0.10 ^a^	3.60 ± 0.13 ^c^
**TFR7**	5.12 ± 0.10 ^bc^	3.67 ± 0.17 ^c^
**CFR7**	5.44 ± 0.20 ^a^	3.50 ± 0.15 ^c^
**TFR15**	4.96 ± 0.25 ^c^	3.51 ± 0.12 ^c^
**CFR15**	5.31 ± 0.20 ^b^	3.41 ± 0.12 ^c^
**TFR30**	4.91 ± 0.20 ^c^	3.40 ± 0.10 ^c^
**CFR30**	5.21 ± 0.27 ^b^	2.30 ± 0.13 ^d^

The values represent the mean ± standard deviation (SD), *n* = 3. Means in columns with different letters differ significantly (*p* ≤ 0.05). TF = Nixtamal flour made with the traditional method, CF = Nixtamalized corn flour made with the industrial (commercial) method, TF0= traditional corn tortilla flour without refrigerated storage, CF0 = commercial corn tortilla flour without refrigerated storage, TFR(7, 15, 30) = traditional corn tortilla flours with refrigerated storage (4 °C) during 7, 15 and 30 days, respectively, CFR(7, 15, 30) = commercial corn tortilla flours with refrigerated storage (4 °C) during 7, 15 and 30 days, respectively.

**Table 5:** Thermal properties of nixtamalized corn flours and corn tortilla flours obtained by the traditional and industrial methods.

Sample	Endotherm 1 (Gelatinization)				Endotherm 2		Endotherm 3	
	To_gel_ (°C)	Tp_gel_ (°C)	Tf_gel_ (°C)	ΔH_gel_ (J/g)	To_RS5I_ (°C)	ΔH_RS5I_ (J/g)	To_RS5II_ (°C)	ΔH_RS5II_ (J/g)
**TF**	70.23 ^a^	78.15 ^a^	80.17 ^a^	5.30 ^a^	102.20 ^f^	6.31 ^d^	112.42 ^e^	10.07 ^g^
**CF**	65.19 ^b^	67.30 ^b^	69.28 ^b^	4.04 ^b^	100.20 ^f^	5.19 ^e^	107.18 ^f^	8.69 ^h^
**TF0**	64.14 ^b^	65.98 ^c^	69.28 ^b^	4.19 ^b^	103.66 ^e^	7.50 ^c^	124.17 ^d^	12.63 ^f^
**CF0**	63.22 ^b^	64.64 ^c^	67.02 ^c^	3.19 ^c^	102.30 ^f^	5.84 ^d^	126.47 ^d^	12.87 ^f^
**TFR7**	62.21 ^c^	65.15 ^c^	68.83 ^b^	2.76 ^d^	107.54 ^d^	8.45 ^c^	130.83 ^c^	16.25 ^d^
**CFR7**	58.89 ^d^	60.19 ^e^	63.08 ^c^	2.14 ^d^	104.65 ^e^	6.20 ^d^	128.34 ^d^	14.64 ^e^
**TFR15**	60.98 ^c^	62.99 ^d^	66.27 ^c^	1.70 ^e^	111.33 ^c^	10.24 ^b^	133.81 ^b^	19.92 ^c^
**CFR15**	57.68 ^d^	59.15 ^e^	61.18 ^d^	1.38 ^e^	110.79 ^c^	7.66 ^c^	130.19 ^c^	17.43 ^d^
**TFR30**	60.36 ^c^	62.75^d^	64.20 ^c^	0.84 ^f^	116.04 ^a^	11.32 ^a^	136.97 ^a^	23.87 ^a^
**CFR30**	56.64 ^d^	58.61^e^	60.34 ^d^	0.73 ^f^	114.25 ^b^	9.34 ^b^	135.91 ^a^	21.84 ^b^

The values represent the mean ± standard deviation (SD), *n* = 3. Means in columns with different letters differ significantly *(p* ≤ 0.05). To_gel_ = Onset gelatinization temperature, Tp_gel_ = peak gelatinization temperature, Tf_gel_ = final gelatinization temperature, ΔH_gel_ = gelatinization enthalpy, To_RS5I_ = peak temperature of melting of the amylose–lipid complexes Type I, ΔH_RS5I_ = melting enthalpy of the amylose-lipid complexes Type I, To_RS5II_ = peak temperature of melting of the amylose–lipid complexes Type II, ΔH_RS5II_ = melting enthalpy of the amylose–lipid complexes Type II.
